# Honey Bee Colonies Headed by Hyperpolyandrous Queens Have Improved Brood Rearing Efficiency and Lower Infestation Rates of Parasitic *Varroa* Mites

**DOI:** 10.1371/journal.pone.0142985

**Published:** 2015-12-21

**Authors:** Keith S. Delaplane, Stéphane Pietravalle, Mike A. Brown, Giles E. Budge

**Affiliations:** 1 Department of Entomology, University of Georgia, Athens, Georgia, 30602, United States of America; 2 Food and Environment Research Agency, Sand Hutton, York, YO41 1LZ, United Kingdom; 3 Animal and Plant Health Agency, Sand Hutton, York, YO41 1LZ, United Kingdom; University of North Carolina, Greensboro, UNITED STATES

## Abstract

A honey bee queen mates on wing with an average of 12 males and stores their sperm to produce progeny of mixed paternity. The degree of a queen’s polyandry is positively associated with measures of her colony’s fitness, and observed distributions of mating number are evolutionary optima balancing risks of mating flights against benefits to the colony. Effective mating numbers as high as 40 have been documented, begging the question of the upper bounds of this behavior that can be expected to confer colony benefit. In this study we used instrumental insemination to create three classes of queens with exaggerated range of polyandry– 15, 30, or 60 drones. Colonies headed by queens inseminated with 30 or 60 drones produced more brood per bee and had a lower proportion of samples positive for *Varroa destructor* mites than colonies whose queens were inseminated with 15 drones, suggesting benefits of polyandry at rates higher than those normally obtaining in nature. Our results are consistent with two hypotheses that posit conditions that reward such high expressions of polyandry: (1) a queen may mate with many males in order to promote beneficial non-additive genetic interactions among subfamilies, and (2) a queen may mate with many males in order to capture a large number of rare alleles that regulate resistance to pathogens and parasites in a breeding population. Our results are unique for identifying the highest levels of polyandry yet detected that confer colony-level benefit and for showing a benefit of polyandry in particular toward the parasitic mite *V*. *destructor*.

## Introduction

The honey bee queen (*Apis mellifera* L) mates with many males, stores their sperm in her spermatheca, and thereafter uses the sperm to fertilize her life’s output of eggs. This habit, called polyandry, results in daughters of mixed paternity, with the number of patrilines in a colony potentially equaling the number of males represented in the queen’s spermatheca. Field studies have shown a positive relationship between a queen’s expression of polyandry and measures of her colony’s fitness, including population growth, weight gain [1], and survival [2]. Numerous hypotheses have been put forward to explain colony-level adaptive benefits of this behavior: Polyandry (1) provides for beneficial non-additive interactions among subfamilies resulting in a more resilient colony [3,4], (2) increases environmental stability by “averaging out” extreme phenotypes [5], (3) reduces variance in the production of diploid males (which are sterile and normally killed by workers) [6], and (4) increases genetic variation within colonies, reducing the likelihood that parasites or pathogens will decrease worker populations to a point that jeopardizes colony survival and reproduction [7]. It was Keller and Reeve [8] who pointed out that all these constitute variations on a single genetic variability (GV) hypothesis in distinction to other contenders such as sperm dose [9,10] or genetic correlations between mating frequencies of males and females [11]. And although none of these hypotheses is mutually exclusive [4], it is safe to say that the GV hypothesis holds a prominent place in theory and experimental support.

In the case of hypothesis (4), support has come in a string of papers arguing that parasites and pathogens are among the most powerful selectors favoring polyandry in social insects [12,13,14,15]. Field studies have shown a positive relationship between polyandry and a colony’s resistance to the diseases chalkbrood and American foulbrood [16,17,18].

But in the case of rare specialist alleles such as those conferring disease or parasite resistance, a simple GV model for polyandry’s benefits may require more nuance. Theory shows that over 90% of the change in within-colony genetic relatedness is explained by a queen’s first 6 matings [12], but honey bee queens routinely reach beyond this asymptote and mate, on average, with 12.0 ± 6.3 males [19] and some up to 40 [20]. At a superficial level it looks like the more the better–but not for the simple reason of greater within-colony variation as shown by Palmer and Oldroyd [12]. Rather, it has been suggested that benefits of polyandry accrue from a queen capturing more of the relatively rare resistance alleles in a breeding population [21]; therefore, we propose a modification of hypothesis (4) under the influence of (5) a rare allele model.

In the context of genetic resistance to novel parasites, the rare allele model may be of key importance and begs the question of the constraints limiting higher mating numbers in nature. These constraints are thought to be risks to the queen from sexually-transmitted diseases [22] and hazards associated with repeated mating flights. But these constraints may be modified under selection by a sufficiently virulent parasite for which there are few resistance alleles.

The mite *Varroa destructor* Anderson and Trueman provides a model of such a virulent parasite. On its newest host *A*. *mellifera* this mite triggers multiple pathologies at the level of individual bee and colony. Left untreated, most managed colonies in temperate zones die within 3–4 years of initial infestation [23]. Being a non-natural parasite, *V*. *destructor* also provides a good system for testing a rare allele model because *A*. *mellifera* has relatively few resistance alleles for it.

A first step in analyzing the rare allele model is to confirm if high degrees of polyandry do in fact confer benefit against a model parasite such as *Varroa*. In this study we created three classes of honey bee queens with exaggerated ranges of polyandry by instrumentally inseminating each with semen from 15, 30, or 60 drones. We measured colonies headed by these queens for various fitness indicators including presence of *V*. *destructor*. Our purpose was to identify lower ranges of effective polyandry in the context of this highly damaging parasite. Moreover, given the ongoing honey bee health crisis, it is important to examine the extent to which benefits of polyandry can be delivered to managed systems.

## Materials and Methods

### Queens and Insemination Procedure

This experiment was conducted in the summers of 2012 and 2013 at research apiaries maintained by Fera (North Yorkshire, UK). Each year, sister virgin queens were reared from one common mother and emerged and maintained individually in polystyrene mating nuclei (Apidea Vertriebs AG, Switzerland) during and after the insemination procedure. Each virgin queen was instrumentally inseminated with semen of 15 drones, 30 drones, or 60 drones (see resulting *n* below). Because our independent variable was polyandry and not genetic variation *per se*, we wanted to narrow the range of genetic variation available from our drone pool. We did this each year by identifying 15 pre-existing colonies to serve as drone sources, their number chosen to match the multiples in our experimental design. Queens inseminated with 15 drones received semen from one drone from each of the 15 colonies; queens receiving 30 drones got two drones per source colony, and queens receiving 60 drones got four drones per source colony. Queens were instrumentally inseminated with standard methods [24,25], including constant care of queens by nurse bees in nucleus colonies before and after insemination and supplementary 1-min treatments of CO2 the day after insemination (CO2 stimulates egg-laying [26]). Polyandry was achieved by collecting semen from the target number of drones into one capillary tube, expelling the contents into a small vial, adding an equal volume of sterile physiological saline, manually mixing the contents, then drawing it back into the capillary tube for subsequent inseminations [24]; admixture with saline was done to promote homogenous expression of drones within each treatment group. The insemination volume for every queen was fixed at 8 μl [25] from the pooled, homogenized sample.

### Experimental Colonies

Each year one dedicated apiary was established, each colony of which comprised of *ca* 1 kg adult bees, three frames of brood of mixed ages, and two combs of honey. Colonies were established in single brood-chamber British national hives, fed supplemental sugar solution and protein as necessary, and provisioned with honey supers as needed to accommodate incoming nectar collected by foragers. Each colony was assigned one of the three experimental treatments: (1) queen inseminated with semen of 15 drones, (2) 30 drones, or (3) 60 drones. After inseminated queens were confirmed as egg-layers in their respective nuclei, each was caged, placed into one of the full-sized field colonies, and released after 1–3 days. After all queens were released, all colonies were maintained for another 6 weeks to ensure adequate queen egg-laying performance and to allow time for incipient worker bee populations (of uncontrolled genetic backgrounds) to die off. This resulted in beginning treatment replicate numbers of (1) 15 drones: *n* = 6_2012_ and 5_2013_, (2) 30 drones: *n* = 8_2012_ and 5_2013_, or (3) 60 drones: *n* = 9_2012_ and 4_2013_.

### Dependent Variables

Each year after week 6 we sampled surviving colonies to determine worker bee population, brood production, daily comb construction, and presence of *Varroa* mites. For colonies set up in 2012, samplings were made on 25 Jul 2012, 13 Aug 2012, 30 Aug 2012, 26 Sep 2012, 1 Nov 2012, 3 Dec 2012, 7 Jan 2013, 5 Feb 2013, 27 Mar 2013, 2 Apr 2013, 26 Apr 2013, 5 Jun 2013, and 20 Jun 2013. For colonies set up in 2013, samplings were made on 14 Aug, 27 Aug, and 4 Sep. It was not possible to measure every dependent variable on every sampling date.

Worker bee population and brood production were derived by visually summing proportions of whole deep frames covered by workers or brood [27], converting frames of adult bees to bee populations with the regression model of Burgett and Burikam [28], and converting frames of brood to cm^2^ by the observation that one deep British National comb (both sides) = 1663 cm^2^.

Comb construction (cm^2^ per day) was determined during periods of nectar flow following the methods of Matilla and Seeley [1]. A honey frame was inserted into the honey super of each hive, each frame provisioned with a 2.5 cm-wide starter strip of beeswax foundation across the top bar. Two days later the frame was removed and the area of foundation drawn into natural comb determined with the visual summing method described above for bees and brood.

Brood production and comb construction were determined relative to worker bee population (per 100 bees) because adjusting strength variables to bee population gives insight into the relative efficiency with which collective tasks in the colony are undertaken; we predict that increasing polyandry improves efficiency of task allocation among subfamilies [1,4].

The presence of *Varroa* mites was determined by collecting a sample of *ca* 300 adult bees (and their phoretic mites) from the middle brood combs of each colony, agitating the sample in alcohol, straining it, and counting the number of bees and mites [29]. Mite samplings were made on 25 Jul 2012, 13 Aug 2012, and 4 Sep 2013.

### Statistical Analysis

Brood production and daily comb construction were analysed by the Restricted Maximum Likelihood method (REML) to account for repeated measures on colonies as well as unequal replication among treatments. In this case, the random terms of the model (colony and time point) were used to define a uniform correlation across all time points within each colony. Treatment (level of polyandry) was the fixed effect. When treatment was found to have a significant effect, we separated individual treatments with pairwise comparisons and means separating groups. Predicted treatment means and 95% confidence intervals are presented. In the case of the *Varroa* mite data, we ran a non-parametric test because of a large number of zeros. Data were summarized across all observation points and classified as either containing mites or not. The proportions of observations positive for mites were then compared across the three treatments using a chi-square permutation test. All analyses were carried out using GenStat 17.1 [30].

## Results

Table 1 presents predicted means (for an average colony and time point) and 95% confidence intervals from the REML analysis. The amount of brood (cm^2^) produced per 100 bees was significantly higher in colonies with queens inseminated with 30 or 60 drones than in colonies with queens inseminated with 15 drones (*F* = 5.2; df = 2,26; *P* = 0.013). No effect of insemination treatment was detected for cm^2^ comb constructed per 100 bees per day (*F* = 0.72; df = 2,11; *P* = 0.51). The chi-square permutation test (Table 2) shows that the proportion of samples positive for *Varroa* mites was not the same in all three treatments, with proportion of samples positive for mites higher than expected in colonies with queens inseminated with 15 drones.

**Table 1:** Performance measures of honey bee colonies whose queens were inseminated with pooled semen from 15, 30, or 60 drones. Values are predicted means ± 95% CI. Means with different letters are different at the 5% significance level.

Treatment	Lower	Predicted mean	Upper
Brood production (cm^2^ per 100 worker bees)			
15	25.2	33.2 a	41.2
30	38.4	44.8 b	51.2
60	42.7	48.6 b	54.5
Daily comb construction (cm^2^ per 100 worker bees)			
15	0.38	0.77	1.16
30	0.67	1.06	1.45
60	0.57	1.00	1.44

**Table 2:** Chi-square contingency test for number of samples positive or negative for *Varroa* mites (Pearson’s *χ* ^2^ = 9.12; range of 1000 permutations: 0.13–13.31; *P* = 0.015).

Treatment	Actual values		Predicted values (under the assumption of identical ratio of positive to negative samples in all three treatments)	
	Negative	Positive	Negative	Positive
15	9	8	13.3	3.7
30	19	2	16.4	4.6
60	19	3	17.2	4.8

## Discussion

Our results add to a growing base of evidence showing adaptive benefits of polyandry to the honey bee colony. Among colonies whose queens were inseminated with 15, 30, or 60 drones we found significantly more brood per bee and a lower proportion of samples positive for *Varroa* mites in colonies whose queens were inseminated with 30 or 60 drones. Under a simple GV model, the rapid plateauing of within-colony genetic relatedness with a queen’s first 6 matings [12] would lead us to predict that no real differences exist within the range of polyandry used in this experiment (15–60 males). However, compared to a paternity number of 15, paternity numbers ≥30 showed significant improvements in two important fitness metrics which suggests benefits of polyandry at rates notably higher than the species average for *A*. *mellifera* (~12 males [19]).

Our results are unique for identifying the highest levels of polyandry yet detected (*n*≥30 males) that confer colony-level benefit and for showing a benefit of polyandry in particular toward the parasitic mite *V*. *destructor*. By expanding the upper range of known queen mating number associated with colony benefits, our results underscore the importance of understanding how natural selection can reward polyandry at levels significantly higher than the species average. We believe two models are plausible starting points:

1. Our results are consistent with a model proposing beneficial non-additive interactions among subfamilies (hypothesis (1) in Introduction). Indeed, in those cases documenting natural evolution of *Varroa* resistance in managed *A*. *mellifera* there is invariably more than one resistance character involved [31,32]. To the extent these characters are under genetic control, parsed among subfamilies, and interact favorably this condition would select for extreme polyandry.
2. Our results are also consistent with a rare allele model predicting that high rates of polyandry are associated with lower infestation rates of a virulent parasite such as *Varroa* for which there are few resistance alleles. The fact that *Varroa* resistance in *A*. *mellifera* is under partial genetic control is well-documented (see review in Rosenkranz et al [23]), but being a non-natural parasitic relationship these resistance alleles are not numerous. This allelic poverty been shown for such mite-resistance phenotypes as hygienic behavior [33], reduced mite reproduction [34], and bee grooming behavior [35]. *Varroa* has been in the UK only since 1992 [36], further limiting possibilities for resistance mutations. Moreover, it is arguable that the practice of beekeeper-applied acaricides retards accumulation of rare resistance alleles in managed populations. Thus the *Varroa* / *A*. *mellifera* system is a good candidate for testing a rare allele hypothesis for the evolution of honey bee polyandry.

Two field studies give valuable insight into the association of hyperpolyandry and *Varroa* levels. When examining 30 colonies in Germany whose queens were naturally mated with a range of 10–28 males, Neumann and Moritz [37] failed to detect a correlation between colony *Varroa* mite levels and queen mating number. In New York state, Tarpy et al [38] found no significant differences in mating number between queens from feral (therefore untreated for *Varroa*) colonies in the Arnot Forest (mean mating frequency 17.4 ± 9.8) and queens from two nearest managed apiaries (20.8 ± 5.9 or 16.0 ± 6.4). From the vantage of an uncomplicated GV model, neither study supports the expectation that polyandry increases in response to a virulent parasite such as *Varroa*. Indeed, Tarpy et al [38] conclude that “hyperpolyandry of honey bees has been shaped on an evolutionary timescale rather than on an ecological one.” However, neither study constitutes a direct test of the rare allele model with which we expect benefits to accrue from effects that are more stochastic (presence or absence of rare alleles) than additive (GV *sensu stricto*). Indeed, the model seems most promising for explaining polyandry at the extreme highs of known distributions. Our experiment shows benefit at these high ranges. Perhaps we need to rethink what constitutes *hyper*polyandry.

A direct test of the rare allele model would require the disambiguation and control of three potentially interacting factors: the genetic variation represented in the queen’s spermatheca (GV *sensu stricto*), her degree of polyandry, and presence or absence of beneficial alleles.

Finally, it cannot be overlooked that polyandry may have an expanding role to play in mitigating health problems facing managed *A*. *mellifera* around the world. The pursuit of genetic host resistance, traditionally focused on targeted character-based selection programs, is championed as the best sustainable solution to the non-natural *Varroa* / *A*. *mellifera* relationship [23]. But it is not mutually exclusive to integrate traditional character-based selection with enhanced polyandry in order to maximize genetic benefits for managed systems.
